# The Significance of Mylohyoid Muscle Release (MMR) in the Vertical and Horizontal Ridge Augmentation Surgeries; Clinical and Human Cadaver Analyses of the Techniques

**DOI:** 10.3390/genes14030595

**Published:** 2023-02-26

**Authors:** Nikolaos Soldatos, Jessica Immonen, Georgios Romanos, Robin Weltman

**Affiliations:** 1 Ashman Department of Periodontics and Implant Dentistry, NYU College of Dentistry, New York University, 345 E. 24th Street, New York, NY 10010, USA; 2 Department of Periodontics, School of Dentistry, Oregon Health Science University (OHSU), 2730 SW Moody Ave, Portland, OR 97201, USA; 3 School of Dental Medicine, University of Nevada Las Vegas (UNLV), 1001 Shadow Ln., Las Vegas, NV 89106, USA; jessica.immonen@unlv.edu; 4 Department of Periodontics and Endodontics, School of Dental Medicine (SDM), Stony Brook University, 106 Rockland Hall, Stony Brook, NY 11794, USA; 5 Department of Clinical Sciences, School of Dental Medicine, University of Nevada Las Vegas (UNLV), 1001 Shadow Ln., Las Vegas, NV 89106, USA

**Keywords:** mylohyoid muscle release, lingual flap management, vertical ridge augmentation, horizontal ridge augmentation, guided bone regeneration

## Abstract

(1) Background: Ridge augmentations either horizontal (HRA) or vertical (VRA) in the posterior mandible are very challenging regenerative procedures. To attain and retain tension-free primary closure, buccal periosteal and mylohyoid muscle releases should be performed. The purpose of the present study was to review, analyze and discuss the three different techniques for the mylohyoid muscle release (MMR) in VRA and HRA surgeries on a clinical and human cadaver level. (2) Presentation of the techniques: Three different techniques are described in the literature regarding the lingual flap management: (i) the finger sweep technique (FST), (ii) the release of the mylohyoid muscle attachment on the lingual flap (MMALF), and (iii) the mylohyoid preservation technique (MPT) in three key anatomical zones. All three techniques, even though they use a different approach, can achieve similar amount of horizontal and vertical mylohyoid muscle release although MPT showed statistically significant higher flap advancement. The human cadaver analyses revealed that all three techniques are considered safe since they do not approximate vital anatomical structures. (3) Conclusions: All three techniques are considered safe, but they are not free of limitations or complications; therefore, they should be performed only by highly experienced and trained clinicians. MPT achieved statistically significant higher flap advancement.

## 1. Introduction

Severe cases of periodontal disease, congenital missing teeth, developmental defects, tooth extractions without ridge preservations, odontogenic cysts, tumors, and trauma are some of the etiological findings that can lead to Seibert class I, II, or III defects and require ridge augmentation surgeries [1,2,3,4]. Ridge augmentation can be achieved with the use of distraction osteogenesis, bone blocks, or guided bone regeneration (GBR). GBR has become more predictable approach, with less morbidity for the patients over the years, due to advancement of the surgical techniques and the barrier membranes, and the use of autogenous grafts, allografts, and xenografts [5,6,7,8,9,10,11,12].

Ridge augmentations in the posterior mandible, either HRA or VRA, are very challenging regenerative procedures. The consensus report of the 15th European workshop of Periodontology on bone regeneration and other studies reported that these techniques should be performed by highly experienced and trained clinicians due to complications such as wound dehiscence, membrane exposure, graft exposure, and post-operative infection [12,13,14,15]. Machtei’s findings, presented in a systematic review in 2001, agree with the consensus report since his results showed that membrane exposure yielded a 6-fold greater negative effect on GBR outcomes when compared to guided tissue regeneration (GTR) outcomes [16].

Wound stabilization, adequate blood supply, protection of the underlying blood clot, space maintenance, prevention of the migration of undesired cells from the overlying soft tissue, and tension-free primary closure are principles that should be followed to obtain a successful surgical outcome [13,15,17,18,19,20,21,22,23,24]. Flap tension of 0.01–0.1 N at the time of suturing may result in dehiscence in 10% of the cases, per Burkhardt and Lang in 2010. However, tension more than 0.1 N showed wound dehiscence in 40% of the cases [25].

**Figure 1:**
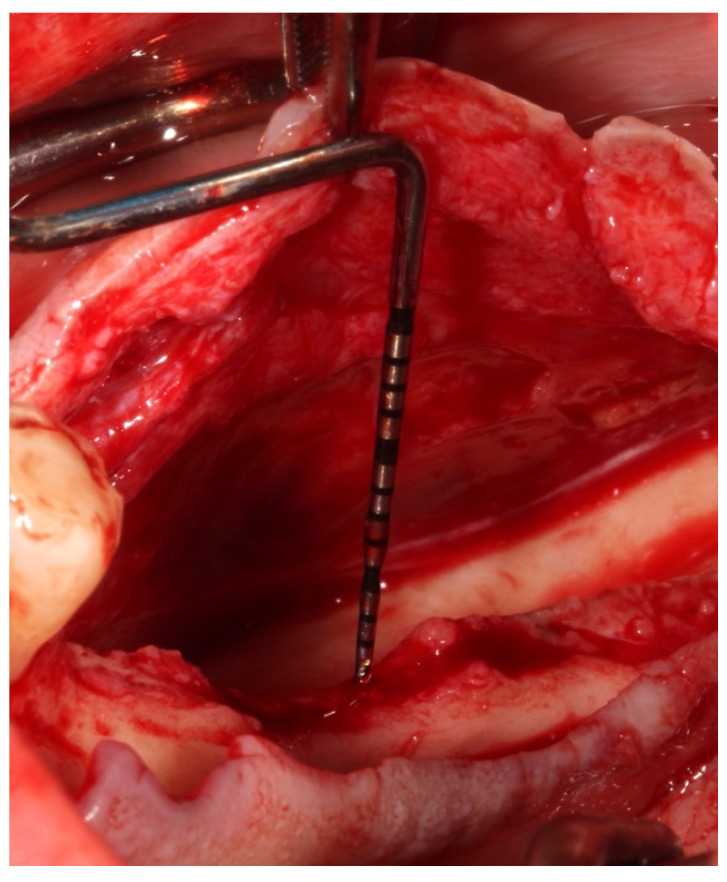
Twenty-three (23) mm of buccal periosteal release in a vertical direction.

**Figure 2:**
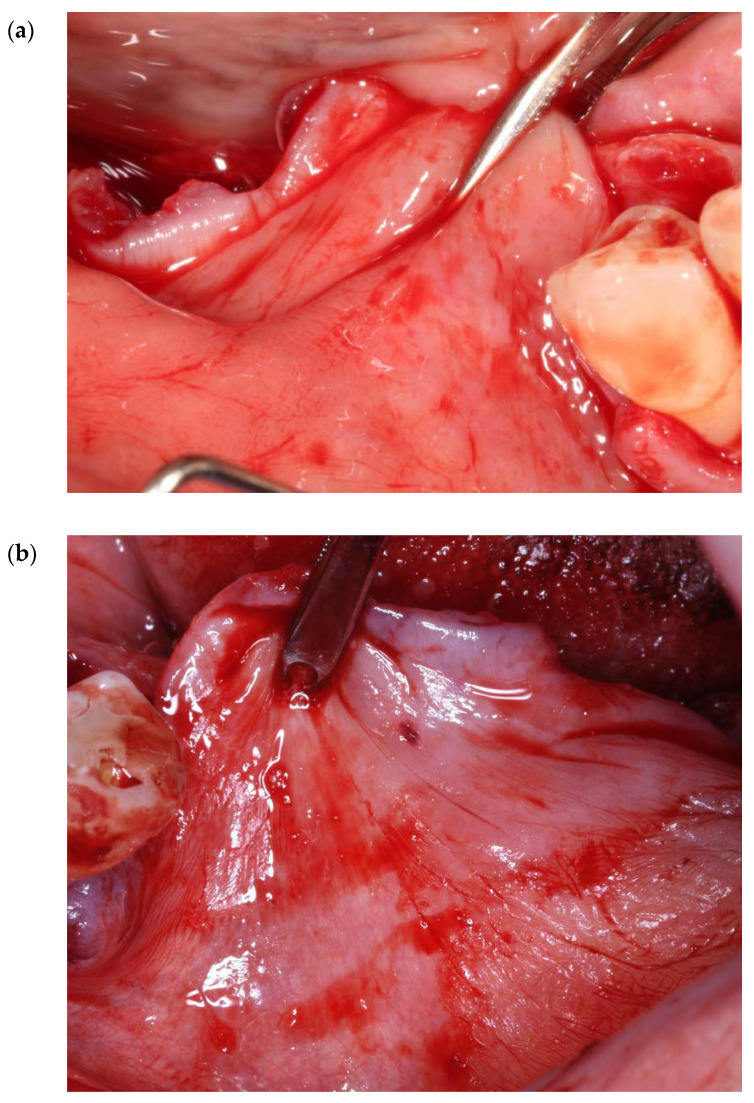
(**a**,**b**) Ten (10) mm of buccal periosteal release in a horizontal direction.

In the posterior mandibular area, to attain and retain tension-free primary closure, buccal periosteal (after isolating the mental nerve) (Figure 1 and Figure 2a,b) and mylohyoid muscle releases should be performed.

**Figure 3:**
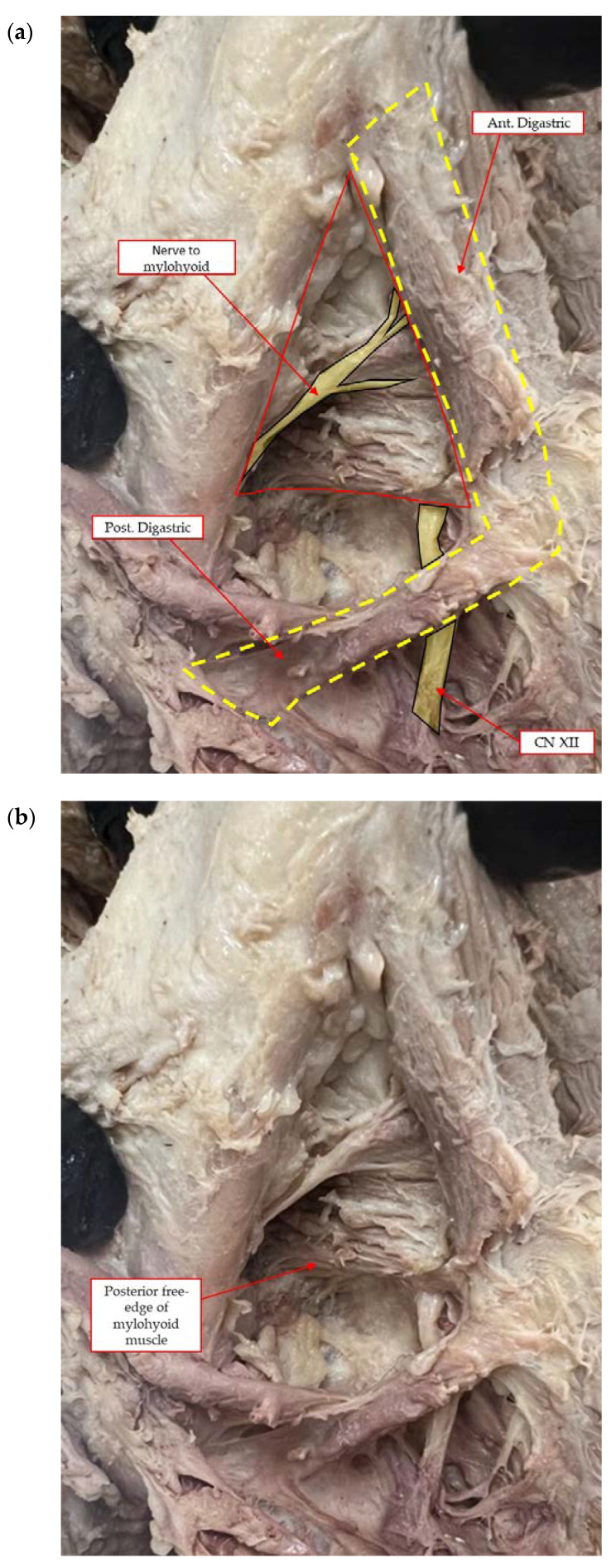
(**a**,**b**) In a human cadaver model, the mylohyoid muscle attachment to the mandible is more apically located in the premolar zone as compared to the attachment at the molar zone. Should the surgical dissection during MMR, extend deeper into the mylohyoid muscle fiber attachment, the vital structures as described above are positioned apical and medial to the mylohyoid muscle.

The mylohyoid muscle or the diaphragma oris muscle is a flat and triangular muscle. It is located superior to the anterior belly of the digastric muscle and forms the floor of the mouth. It is one of the suprahyoid muscles, and it is derived from the first pharyngeal arch. It inserts into the body of hyoid bone and elevates the tongue and the hyoid bone. It is innervated by the inferior alveolar nerve. It has oblique line and runs more superior at the area of the first molar (which is very close to the attachment of the mandible) and then runs deeper at the area of first premolar and anterior teeth (Figure 3a,b) [26].

**Figure 4:**
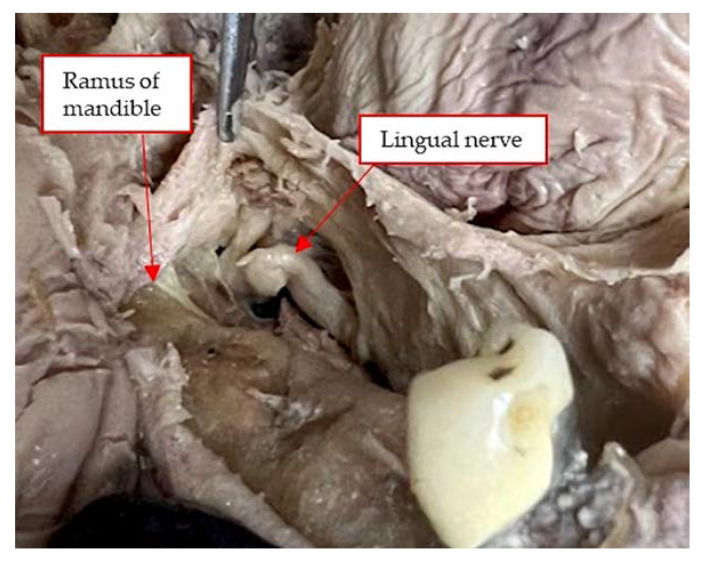
More posteriorly located, the lingual nerve, in a human cadaver, is observed traversing parallel to the ramus dropping apically distal to the location of a third molar.

The posterior portion of the mylohyoid muscle derives from the lingual tuberosity below the retromolar pad [26]. The position of the lingual nerve is shown in the Figure 4 in relation to the design of the lingual flap and the MMR.

**Figure 5:**
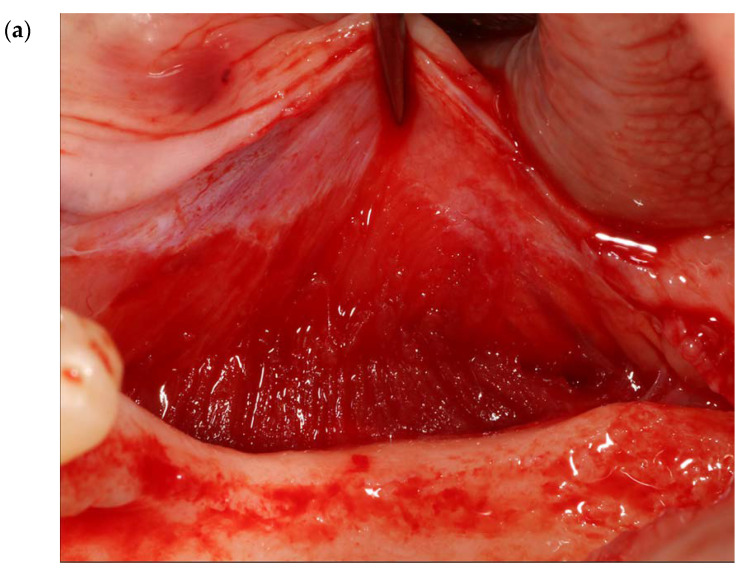

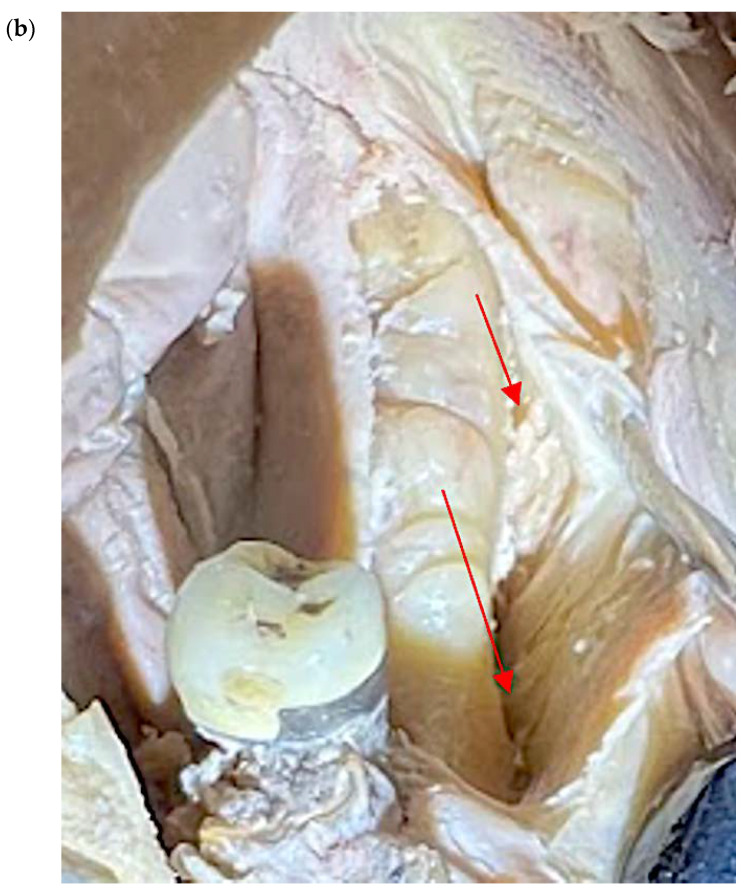
View of the superficial fibers and the attachment of the mylohyoid muscle to the mandible, forming the floor of the mouth: clinically (**a**) and in a human cadaver model [(**b**)-red arrows].

The mylohyoid muscle separates the sublingual from the submandibular space. These spaces are united in case of odontogenic infections penetrating the muscle [14,26]. A very good understanding of the head and neck anatomy is obligatory to avoid severe complications during VRA and HRA surgeries [14] (Figure 5a,b).

Three different techniques have been described in the literature regarding the lingual flap management using the MMR (Table 1): (i) Finger sweep technique (FST), (ii) the release of the mylohyoid muscle attachment on the lingual flap (MMALF), and (iii) the mylohyoid preservation technique (MPT) in three key anatomical zones [13,15,22,27].

The purpose of the present study was to review/analyze/discuss the three techniques for the release of the mylohyoid muscle in VRA and HRA surgeries in partial edentulous and atrophic posterior mandibular areas, in a clinical setting and in a human cadaver model.

## 2. Presentation of the Techniques

### 2.1. Finger Sweep Technique (FST) [19,22,27]

**Figure 6:**
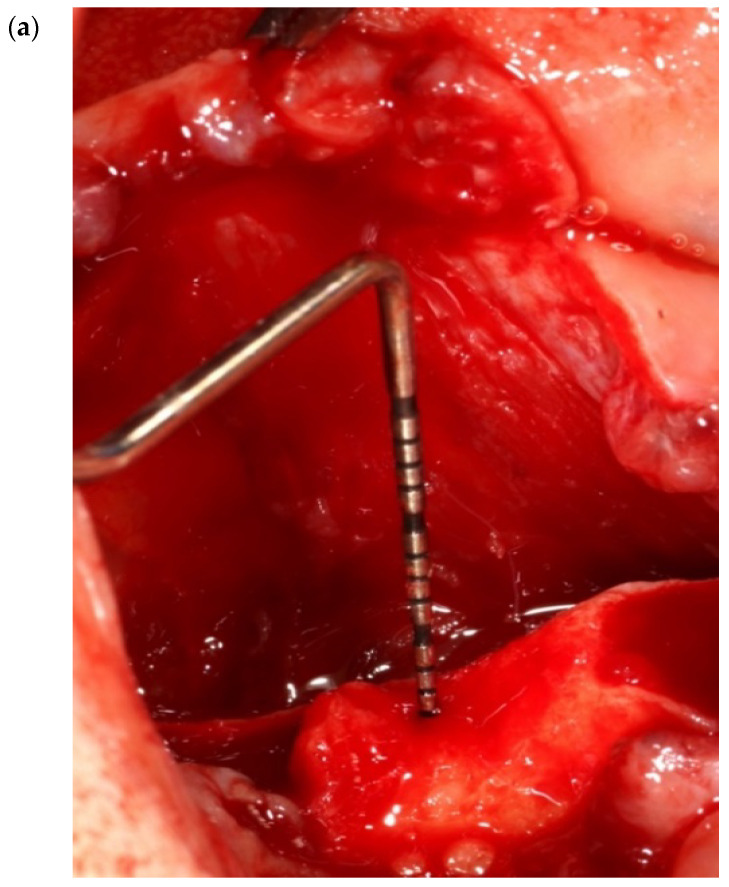

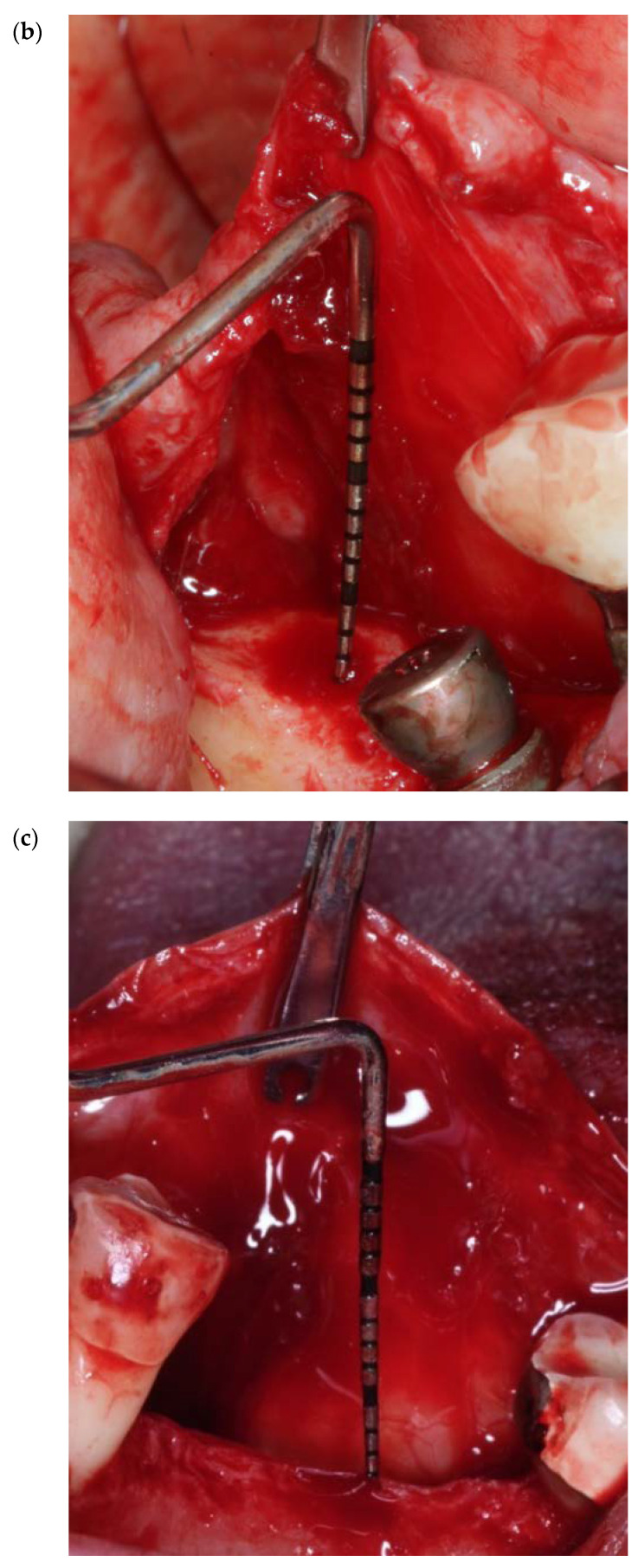
(**a**–**c**) Clinical view of MMR in a vertical direction, ranging from 29–32 mm.

**Figure 7:**
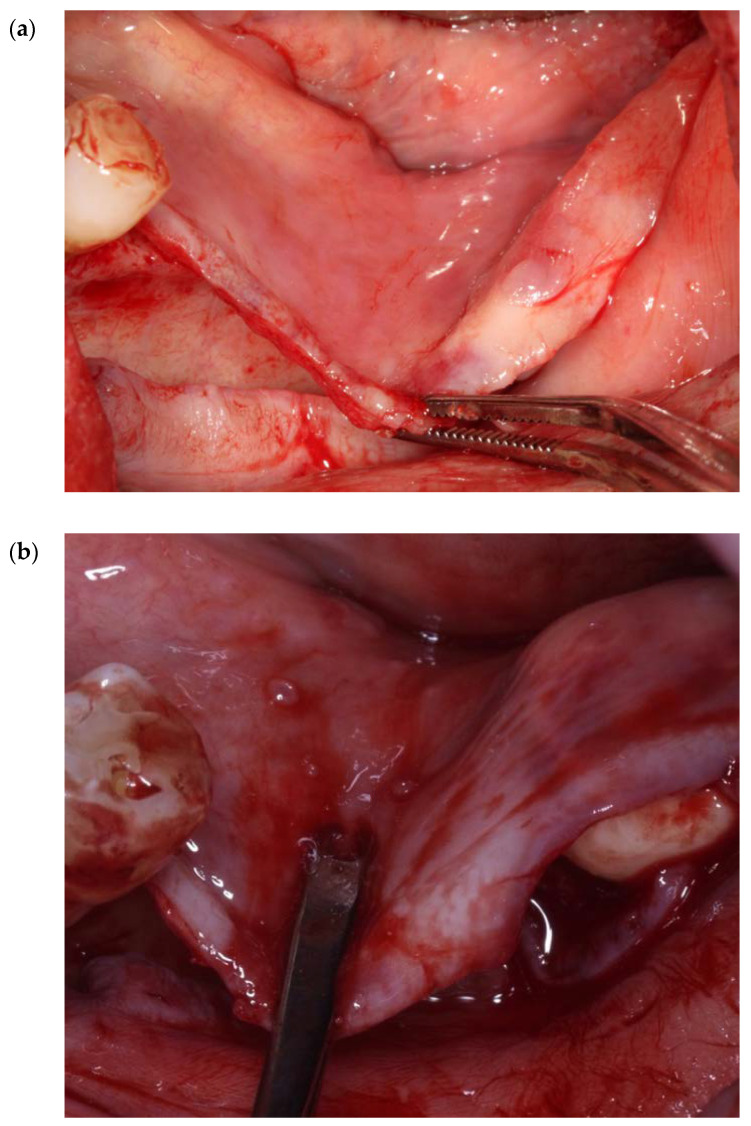

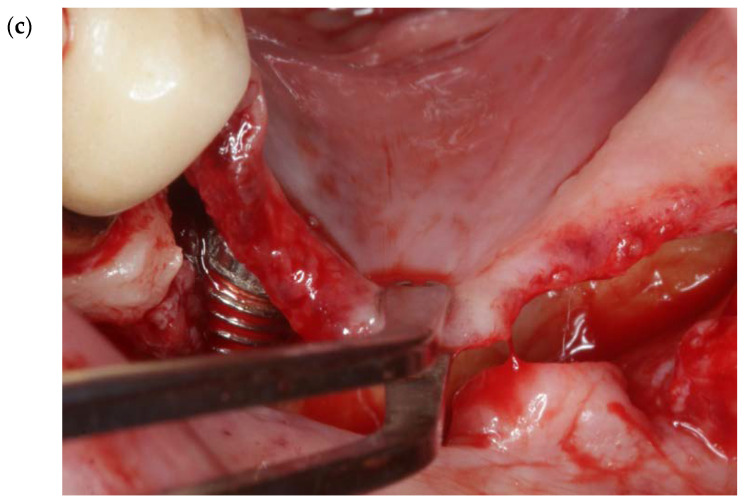
(**a**–**c**) Six to ten (6–10) mm of MMR in a horizontal direction.

**Figure 8:**
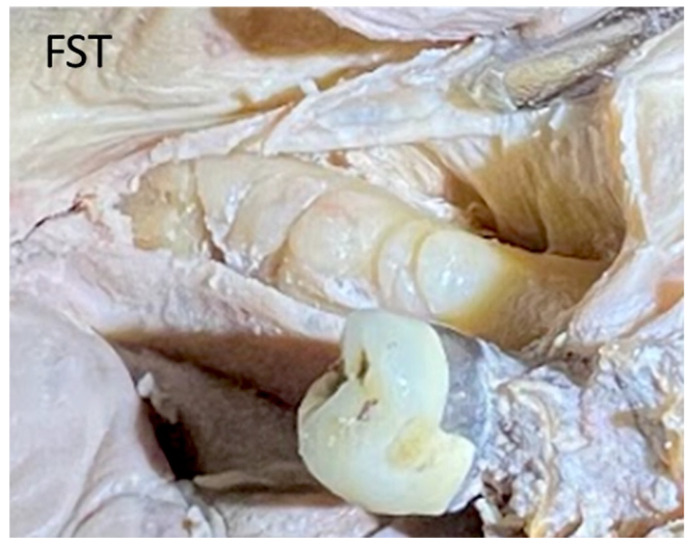
The design of the lingual flap of the FST in a human cadaver model, showing the release of the superficial fibers of the mylohyoid muscle.

**Figure 9:**
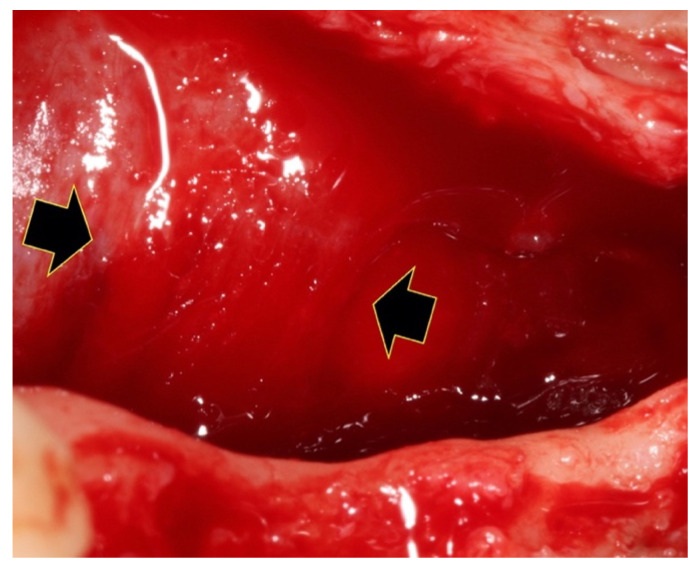
Clinical overview of the connective tissue band of 1–2 cm at the area of the first molar (black arrows), where the mylohyoid muscle attaches to the lingual flap.

**Figure 10:**
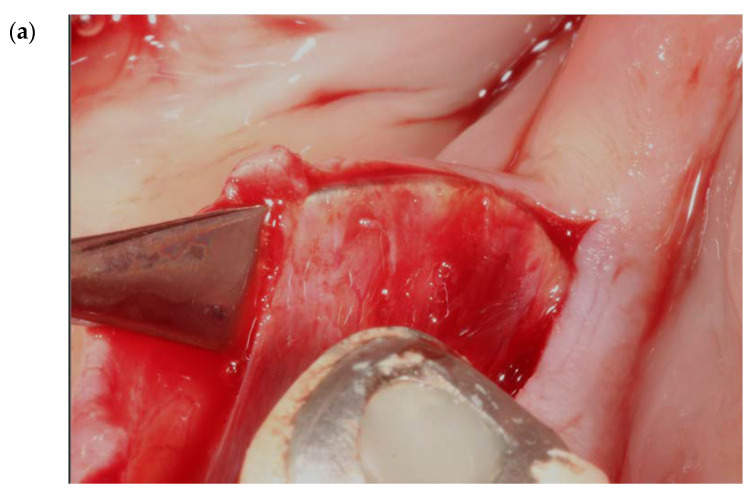

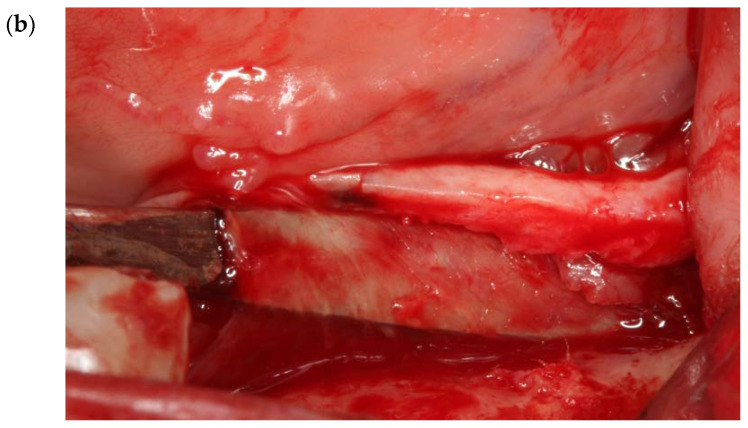
(**a**,**b**) Clinical overview of the placement of two different blunt instruments under the attachment of the mylohyoid muscle at the lingual flap. The connective tissue attachment ranges from 1–2 cm.

The flap design includes a full-thickness reflection of the buccal and lingual flaps, starting from a hockey stick releasing incision at the area of lateral incisors to the retromolar pad and ending in a 1 cm releasing incision to the ramus. FST is a blunt dissection/separation of the superficial from the deep mylohyoid muscle fibers. This finger dissection (using the index finger) includes stripping of the superficial fibers of the mylohyoid muscle in both an anterior and a posterior direction. No sharp dissection is needed. With the FST, the lingual flap release could reach up to 32 mm from the crest in a vertical direction (Figure 6a–c) and 6–10 mm in a horizontal direction towards the buccal aspect (Figure 7a–c and Figure 8).

### 2.2. Release of the Mylohyoid Muscle Attachment on the Lingual Flap (MMALF) [15,28]

The flap design is similar with the FST (please see the description above). The mylohyoid insertion in the lingual flap is a connective tissue band (1–2 cm width) continuing from the epimysium of the mylohyoid muscle, around the area of the first molar (Figure 9).

**Figure 11:**
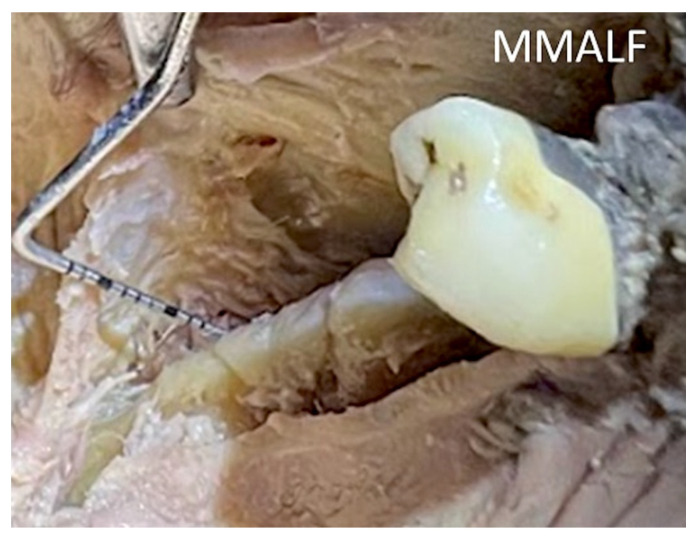
A human cadaver overview of a 23 mm release of the mylohyoid muscle using the MMALF technique.

A blunt instrument (e.g., Prichard elevator) is inserted under this connective tissue band, and the insertion of the muscle in the lingual flap is detached with a gentle traction in a coronal direction (Figure 10a,b and Figure 11).

This technique allows for a partial detachment of the mylohyoid muscle. Like the FST, the lingual flap release could reach more than 30 mm from the crest in a vertical direction and 6–10 mm in a horizontal direction towards the buccal aspect.

### 2.3. Mylohyoid Preservation Technique (MPT) in Three Key Anatomical Zones [6,13,14]

The flap design in this technique consists of a full-thickness reflection extending distally within 2 mm of the retromolar pad. Then a distal oblique vertical incision is made towards the coronoid process of the mandible. A vertical incision is made, mesial and buccal, two teeth away from the area of anticipated regeneration. Mesial and lingual, a 3–4 mm incision is performed at the mesial lingual line angle of the most distal tooth in front of the area of regeneration.

**Figure 12:**
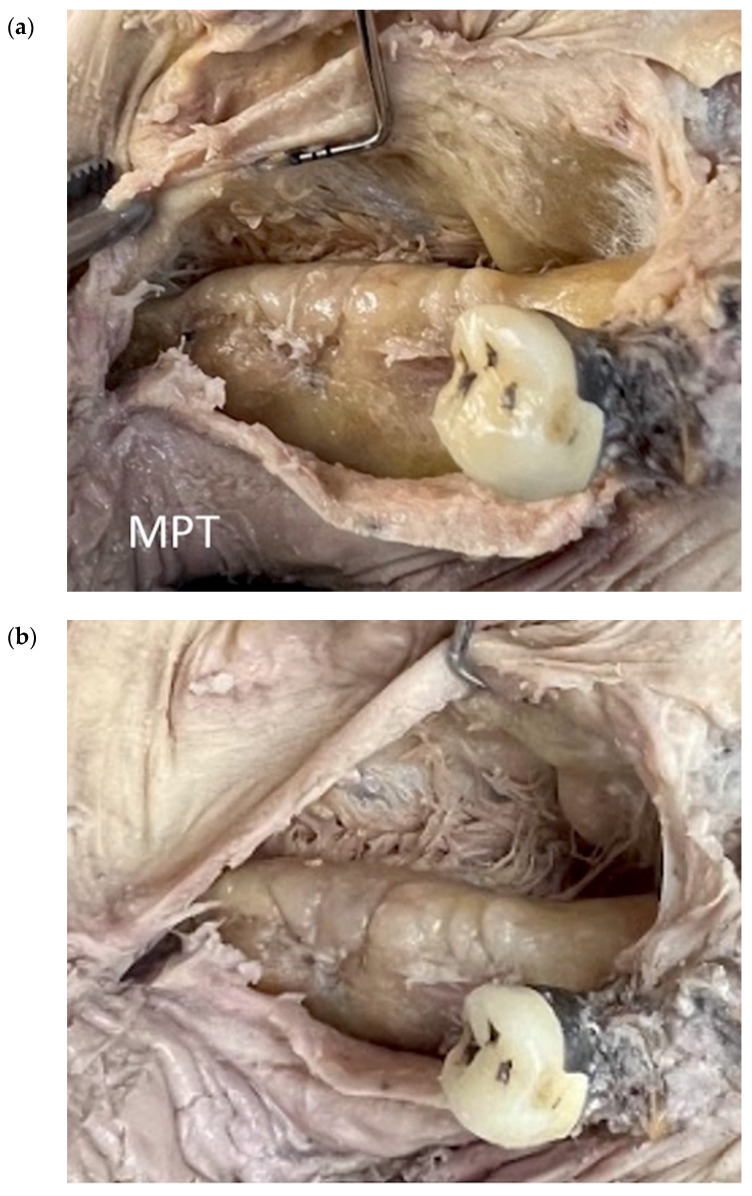
MMR using the MPT technique in a human cadaver model. (**a**) The soft tissue superior to the muscle insertion is pushed in a lingual direction (zone II). (**b**) In the anterior section of the lingual flap, a semi-blunt periosteal release is performed in a sweeping motion with a 15c blade (zone III).

This technique considers three key anatomical zones: (i) tunneling and lifting of the retromolar pad (zone I), with the use of a periosteal elevator, the retromolar pad is reflected and pulled in a coronal direction; (ii) flap separation with mylohyoid muscle preservation (zone II, Figure 12a), as the soft tissue superior to the muscle insertion, with the use of a blunt instrument, is pushed in a lingual direction; and (iii) anterior, semi blunt periosteal release (zone III, Figure 12b), in which, with the use of a 15c blade in a perpendicular angle, an incision is performed in a sweeping motion. The goals of this technique are to include the retromolar pad into the lingual flap, which allows for maximum flap release; to separate the flap from the superior muscle fibers; and to provide flap flexibility, which will prevent any wound dehiscence. Similar to the previously discussed techniques, the lingual flap release could reach more than 30 mm from the crest in a vertical direction and 6–10 mm in a horizontal direction towards the buccal aspect.

## 3. Cadaver Analyses of the Techniques

Blunt dissection with finger pressure, as described with the FST, thins the periosteal layer to expose and release the immediately underlying superficial mylohyoid muscle fibers. More posteriorly located, the lingual nerve is observed traversing parallel to the ramus, dropping apically distal to the location of a third molar. The MMALF technique describes initial penetration through the periosteum (at the level of the first molar) within the superior layers of the mylohyoid fiber attachment. Vital structures including the lingual nerve, artery, and submandibular salivary ducts are located apical and medial to the dissection. The MPT describes a semi-blunt penetration within the periosteum at the premolar zone. The mylohyoid muscle attachment to the mandible is more apically located in the premolar zone as compared to the attachment at the molar zone. All three techniques are considered safe (provided that the clinicians are experienced and properly trained) since they do not approximate any vital structures located below the mylohyoid muscle.

## 4. Discussion

It is of paramount importance to follow the prescribed surgical principles for buccal and lingual flap management to predictably attain and maintain tension-free primary closure during the healing period and avoid any post-operative complications. During the lingual flap management, severe complications may occur, including the risk of damaging the lingual nerve, the sublingual artery, the Wharton’s duct, the sublingual and submandibular glands, and perforating/overthinning the lingual flap [7,14,15,28,29,30]. These events may result in hematoma, which can be a life-threatening complication due to possible respiratory obstruction [31].

Urban et al. compared in a fresh human cadaver study FST and MMALF (control groups) with MPT (test group) [13]. The authors did not explain how many specimens in the control group were divided between the FST and the MMALF. Between the control and test groups, the test group technique showed statistically significant higher flap advancement in all three zones [13]. However, there is no study in the literature directly comparing the three techniques.

Ronda et al. in 2011, performed MMALF in a study with 69 VRA surgeries with the consequent placement of 187 implants [15]. The study reported four sites with signs of infections 2 weeks postoperatively, where the implants and the bone grafting materials had to be removed but not related to the lingual flap management. No membrane exposure was observed, or hemorrhagic issues such as hematoma, for the rest of the cases [15]. Ronda et al. in 2014, used in a similarly designed study the same technique in patients with atrophic posterior mandibles. Minor complications were reported regarding the lingual flap management, such as edema and hematoma [28].

Several studies described vertical releasing incisions as part of the management of the lingual flap. Flap retraction and membrane exposure were noted in four cases where the membranes were removed, and no regeneration was achieved [32,33,34]. Some studies did not report any complications with the use of FST [9,19,22,27]. Other studies for VRA did not report the technique was performed regarding the lingual flap management [7,35,36].

All three techniques are not free from limitations and complications. FST may create a communication between the surgical site and the submandibular or sublingual space. In case of infection, a severe medical complication may occur [19,22,27]. Removing the connective tissue band with the use of MMALF may lead to overthinning and necrosis of the lingual flap, which may cause exposure of the graft in the early healing period [15,28]. The MPT is a more complex and technique-sensitive approach, compared to the other two, in addition to the use of a 15c blade over the mesial aspect of the lingual flap, risking a possible perforation [6,13].

Soldatos and Weltman concluded that (i) flap management, with the use of MMR and buccal periosteal release, and (ii) the initial defect morphology are two very important key factors for successful GBR outcomes, especially in VRA [37]. Although flap management is an important principle for GBR procedures, a case report showed 2–5 mm VRA around previously placed dental implants with the use of a dense-PTFE membrane, which was left exposed to heal in a secondary intention [11].

Finally, the mandibular lingual releasing approach of the mylohyoid muscle is used in cases of oral and oropharyngeal carcinomas. The mylohyoid muscle is completely detached to give visual access to the providers [38,39]. In addition, the use of mandibular lingual release was associated with significantly lower frequency of maxillofacial pain and quality of life in these specific patients [39]. To the best of the authors’ knowledge, this is the first study reviewing, analyzing and discussing the three different techniques regarding MMR in VRA and HRA surgeries on a clinical and human cadaver level.

## 5. Conclusions

All three techniques, although using different approaches, achieve similar amounts of horizontal and vertical MMR. The techniques are not free of limitations and complications, due to the proximity to sensitive anatomical areas, but are considered safe, provided that they are performed only by highly experienced and trained clinicians.

## Figures and Tables

**Table 1:** Summary of all three techniques for MMR.

Techniques	Methods	Limitations
FST (Pikos 2005 [22], Romanos 2010 [19])	Blunt dissection/separation of the superficial from the deep mylohyoid muscle fibers without any sharp dissection with the use of the index finger.	Creation of communication between the surgical site and the submandibular or sublingual space.
MMALF (Ronda et al. 2011 [15])	A blunt instrument is inserted under the mylohyoid muscular insertion at the lingual flap. With a gentle traction in a coronal direction, the connective tissue band is detached.	Overthinning of the lingual flap, which may lead to flap necrosis and exposure of the graft in the early healing period.
MPT (Urban et al. 2018 [13])	1. Tunneling and lifting of the retromolar pad (zone I). 2. Flap separation with mylohyoid muscle preservation (zone II). 3. Anterior, semi blunt periosteal release (zone III) with the use of a 15c blade in a perpendicular angle.	More complex and technique sensitive approach, especially in zone III; the use of a 15c blade over the mesial aspect of the lingual flap endangers a possible flap perforation.

## Data Availability

No data were collected or analyzed.
